# Cellular turnover and expression of hypoxic-inducible factor in acute acalculous and calculous cholecystitis

**DOI:** 10.1186/cc6170

**Published:** 2007-10-31

**Authors:** Merja Vakkala, Jouko J Laurila, Juha Saarnio, Vesa Koivukangas, Hannu Syrjälä, Tuomo Karttunen, Ylermi Soini, Tero I Ala-Kokko

**Affiliations:** 1 Department of Anesthesiology, Division of Intensive Care, Oulu University Hospital, Kajaanintie 52, Oulu, Finland, FIN-90029; 2 Department of Surgery, Oulu University Hospital, Kajaanintie 52, Oulu, Finland, FIN-90029; 3 Department of Infection Control, Oulu University Hospital, Kajaanintie 52, Oulu, Finland, FIN-90029; 4 Department of Pathology, Oulu University, Kajaanintie 50, Oulu, Finland, FIN-90029

## Abstract

**Introduction:**

Epithelial corrective and destructive mechanisms have not been studied in inflammatory gallbladder disease.

**Methods:**

Epithelial apoptosis, cell proliferation and expression of hypoxia-inducible factor (HIF)-1α were compared in gallbladders from patients with acute acalculous cholecystitis (AAC; *n *= 30) and acute calculous cholecystitis (ACC; *n *= 21), and from patients undergoing surgery for other reasons (normal gallbladders; *n *= 9), which were removed during open cholecystectomy. The immunohistochemical stains included antibodies to Ki-67 (proliferation), M30 (apoptosis) and HIF-1α. Proliferation and apoptosis were expressed as percentages of positive cells. HIF-1α expression was expressed as absent, weak, or strong.

**Results:**

Apoptosis (median [25th to 75th percentile]) was significantly increased in AAC (1.31% [0.75% to 1.8%], *P *< 0.001) and ACC (1.10% [0.63% to 1.64%], *P *= 0.001), compared with control samples (0.20% [0.07% to 0.45%]. The proliferation rate was significantly increased in AAC (8.0% [4.0% to 17.0%], *P *< 0.001) and ACC (14% [7.5% to 26.5%], *P *= 0.001) compared with control samples (1.0% [1.0% to 3.0%]). Strong HIF-1α staining was observed in 57% of AAC, in 100% of ACC and in 44% of control specimens (*P *< 0.001). Intense HIF-1α expression was associated with increased cell proliferation (*P *= 0.002).

**Conclusion:**

Cell proliferation and apoptosis were increased in AAC and ACC, as compared with normal gallbladders. Expression of HIF-1α was lower in AAC than in ACC.

## Introduction

Acute acalculous cholecystitis (AAC) is an acute inflammation of the gallbladder in the absence of gallstones. It has been diagnosed with increasing frequency in critically ill patients [1-5]. Systemic inflammatory response and disturbances in splanchnic circulation combined with visceral hypoperfusion, and ischaemia-reperfusion injury are assumed to play important roles in the pathogenesis of AAC [6,7]. AAC has also been shown to be associated with multiple organ dysfunction syndrome [6,8]. In contrast, the more common form of acute cholecystitis, namely acute calculous cholecystitis (ACC), is caused by gallstones, which lead to occlusion, distension, oedema, bile stasis and often bacterial infection of the gallbladder [9,10].

Epithelial integrity depends on cell proliferation and cell destruction. The mucosal cell proliferation rate has previously been studied in normal gallbladder mucosa [11]. It has been reported to be low and comparable to that in normal colorectal mucosa [12]. There are no data on the epithelial proliferation rate or apoptosis in inflammatory conditions involving the gallbladder. Apoptosis is (at least in rat intestinal epithelium) the major mode of cell death in ischaemia and ischaemia-reperfusion [13]. Apoptosis is a regulated process, and it is mediated by a sequential cascade of intracellular enzymes [14].

Hypoxia-inducible factor (HIF)-1 is a key factor in the regulation of epithelial integrity [15]. It is a transcription factor that regulates the pathophysiological response to hypoxia and ischaemia by increasing the transcription of various proteins that are involved in angiogenesis, glycolysis, erythropoiesis and cell survival, ensuring cellular function in low-oxygen conditions [16-18]. HIF-1 consists of a constitutively expressed subunit (HIF-1β) and an oxygen-regulated subunit (HIF-1α; or its paralogs HIF-2α and HIF-3α) [15]. No studies of HIF-1 expression in normal or inflammatory gallbladder mucosa have yet been reported. HIF-1 is involved in ischaemia-reperfusion and tumour growth, and its expression is regulated by hypoxia and cytokines or nitric oxide [19,20].

Because the epithelial corrective and destructive mechanisms have not been studied in inflammatory gallbladder disease, we were interested in determining whether these phenomena differ in the main patterns of acute cholecystitis, namely AAC and ACC. Thus, expression of HIF-1α and markers of apoptosis and cell proliferation were compared in these entities and normal gallbladders to elucidate the pathogenesis of these conditions.

## Materials and methods

### Patients

This study was approved by the Ethics Committee of Oulu University Hospital, and informed consent was not required because the data had been collected for clinical purposes and no additional interventions were done. During the years 2000 to 2001, 39 of the 3,984 intensive care unit (ICU) patients treated in this hospital underwent cholecystectomy because of AAC during their ICU stay. The operative finding was necrosis and gangrene in the gallbladder wall in 17 patients (44%) and a thickened gallbladder wall in 22 patients (56%). A detailed report of the clinical and diagnostic features and the outcomes of these 39 patients was published previously [3]. The basic histopathology, including assessment of epithelial necrosis, and epithelial detachment of these gallbladders were also previously described [10].

The AAC group in the present study included 30 randomly chosen patients out of this series of 39. The age (mean ± standard deviation [SD]) of these patients was 60 ± 12.5 years and 19 out of 30 were men. Severity of illness scores on admission (mean ± SD) were as follows: Acute Physiology and Chronic Health Evaluation score II 23.6 ± 6.1, Simplified Acute Physiology Score II 47.2 ± 12.3 and Sequential Organ Failure Assessment score 9.6 ± 3.5. Sepsis (10/30), cardiovascular surgery (8/30) and pneumonia (5/30) were the most common admission diagnoses. The median (25th to 75th percentile) length of ICU stay before cholecystectomy was 7.5 days (2.8 to 15.3 days). Sixteen patients (53.3%) had three or more failing organs at the time of cholecystectomy. Two of the 30 bile samples (6.7%) taken during cholecystectomy were positive for bacterial growth. Hospital mortality was 36.6% (11/30).

The ACC group included 21 consecutive patients undergoing surgery at our hospital during the years 2000 and 2001. An operative finding of an inflamed gallbladder with gallstones was used as the inclusion criterion. All of these patients were admitted to the hospital because of ACC, and none was treated in the ICU. The median (25th to 75th percentile) time from onset of symptoms to surgery was 3 days (2 to 4.5 days), and the median (25th to 75th percentile) time from admission to the hospital to surgery was 2 days (1 to 2 days). The age of these patients (mean ± SD) was 57.9 ± 10.3 years, and six out of 21 were men. Nine out of 16 bile cultures were positive for bacterial growth (56%).

The control group included nine samples taken from normal-looking gallbladders removed during pancreatic tumour resection. These patients had local disease remote from the gallbladder, and they did not have a history of biliary obstruction. The age (mean ± SD) of the patients in the control group was 59.1 ± 17.8 years, and three out of nine were men.

### Immunohistochemical staining

The gallbladder samples were fixed in neutral buffered formalin and embedded in paraffin. Sections (5 μm) were cut from the specimens and placed on glass slides. The sections were stained in haematoxylin and eosin for conventional histopathological diagnosis [10]. The immunohistochemical analyses were conducted in accordance with the manufacturer's recommendations, and they consisted of Ki-67 (proliferation), M-30 (apoptosis) and HIF-1α antibodies. For Ki-67 and M30 stainings, the slides were pretreated with Tris/EDTA (pH 9; 15 minutes). The primary antibodies consisted of a mouse monoclonal Ki-67 antibody (NCL-Ki67-MMl, dilution 1:100, incubation time 30 minutes; Novocastra Laboratories Ltd, Newcastle upon Tyne, UK) and a mouse monoclonal M30 CytoDEATH™ antibody (dilution 1:1,000, incubation time 30 minutes; Roche, Mannheim, Germany). With these two antibodies, the EnVisio kit (Dako, Glostrup, Denmark) was used and the colour was developed with diaminobenzidine. Pretreatment for HIF-1α consisted of 10 mmol/l sodium citrate (pH 6; 10 minutes). The primary antibody was mouse monoclonal HIF-1α antibody (dilution 1:50, at +4°C overnight; Neomarkers, LabVision Corporation, Fremont, CA, USA). The Power Vision kit (Immunovision Technologies, Brisbane, CA, USA) was used for detection, and the colour was developed with diaminobenzidine.

### Evaluation of immunohistochemical staining reactions

Ki-67 positivity was expressed as a nuclear staining pattern. Positive cells were counted in approximately 450 epithelial cells in each sample. The Ki-67 index was expressed as the percentage of Ki-67-positive cells.

M30 detects apoptosis in epithelial cells and is expressed as a cytoplasmic staining pattern. Apoptotic activity, expressed as a M30 index, was determined as the percentage of M30-positive epithelial cells in 10 high-power fields (HPFs) with 40× objective.

HIF-1α expression was assessed in both the cytoplasm and the nucleus by calculating the percentage of positively stained cells. The HIF-1α staining reaction was expressed as absent, weak, or strong, based on the intensity and extent of cytoplasmic and nuclear staining as follows: complete absence of HIF reactivity (score = 0); cytoplasmic reactivity in fewer than 50% of epithelial cells and/or nuclear expression in sporadic cells (<10% of epithelial cells) (score = 1); and cytoplasmic expression in more than 50% of epithelial cells and/or nuclear expression in more than 10% of epithelial cells (score = 2).

All assessments were made by two investigators experienced in immunohistochemistry (MV and either YS or TK).

### Statistical analysis

SPSS 13.0 for Windows (SPSS Inc., Chicago, IL, USA) was used for statistical analysis. The normality of distribution was assessed using the Kolmogorov-Smirnov test. The data are expressed as percentage, as mean ± SD in the case of normally distributed data, and as median (25th to 75th percentile) in the case of non-normally distributed data. Categorical data were analyzed using Fisher's exact test. The Kruskal-Wallis test was used to describe the differences in the percentage of positively stained epithelial cells between the AAC, ACC and control groups. The Mann-Whitney U-test was applied to analyze the differences between two groups (AAC versus control, ACC versus control and AAC versus ACC). Differences were considered significant at *P *< 0.05.

## Results

**Figure 1:**
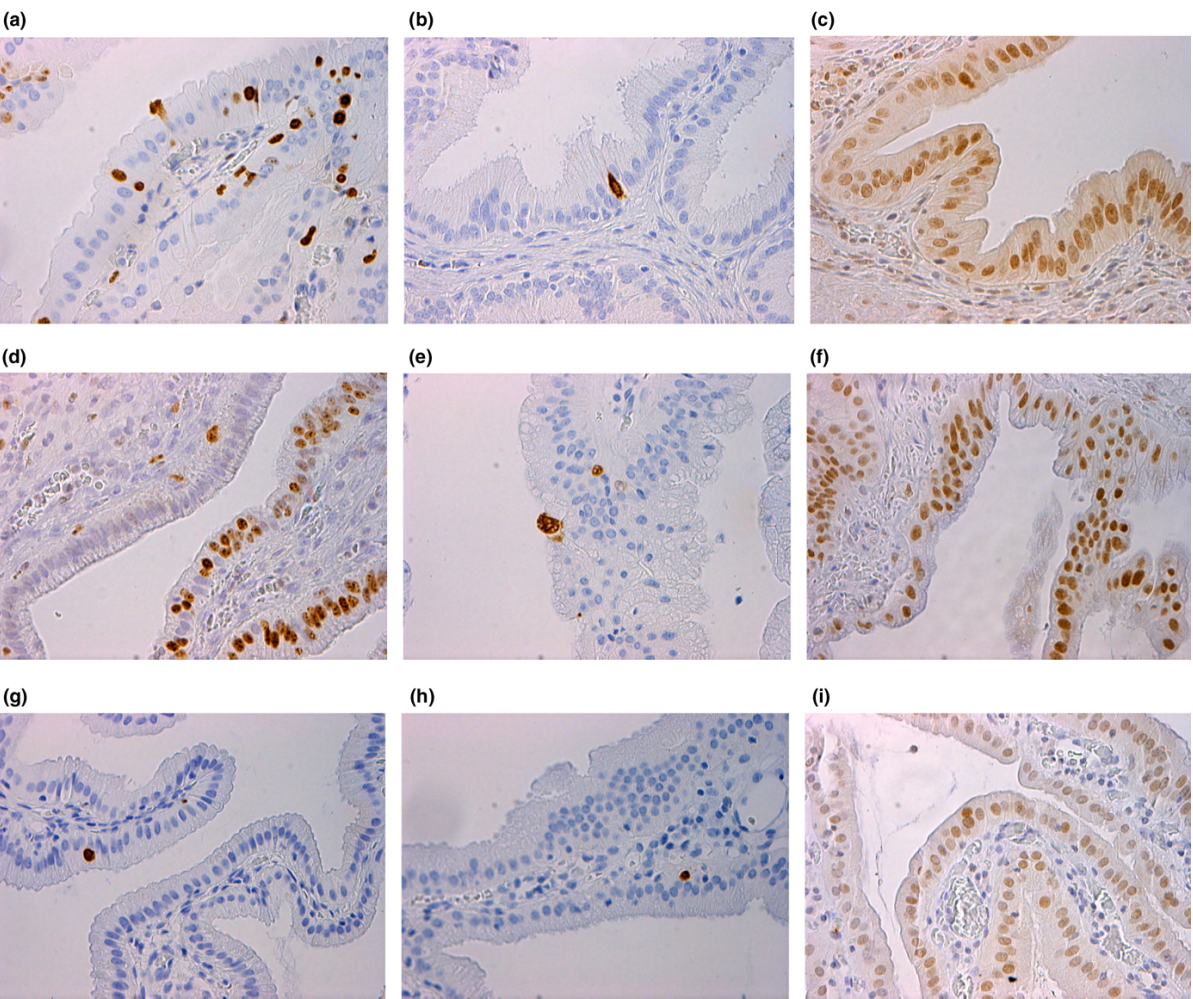
Immunohistochemical staining in AAC, ACC and normal gallbladder mucosa. **(a) **Ki67 expression in acute acalculous cholecystitis (AAC). **(b) **One M30-positive apoptotic cell in AAC. **(c) **Hypoxia-inducible factor (HIF)-1α expression in AAC; nuclear expression and moderate cytoplasmic expression can be seen in this sample. **(d) **Abundant Ki67 expression in acute calculous cholecystitis (ACC). **(e) **two M30-positive cells in ACC. **(f) **strong nuclear expression of HIF-1α in ACC. **(g) **Ki67 in control sample. **(h) **M30-positive cell in control sample. **(i) **Abundant nuclear and weak cytoplasmic expression of HIF-1α in control sample.

Immunoreactivity in AAC, ACC and control samples is shown in Figure 1. Apoptosis (M30 index) was significantly increased in both AAC (1.31% [0.75% to 1.80%], *P *< 0.001) and ACC patients (1.10% [0.63% to 1.64%], *P *= 0.001), as compared with control individuals (0.20% [0.07% to 0.45%]. However, the difference in extent of apoptosis between the AAC and ACC groups was not statistically significant (*P *= 0.50).

Proliferation rate (Ki67 index) was significantly increased in AAC (8.0% [4.0% to 17.0%], *P *< 0.001) and ACC (14.00% [7.5% to 26.50%], *P *= 0.001) compared with control specimens (1.0% [1.0% to 3.0%]. Although higher in ACC than in AAC, the increase in cell proliferation did not reach statistical significance (*P *= 0.196).

Nuclear HIF-1α positivity was higher in the ACC group (80%) than in the control group (20%), and this difference was almost statistically significant (*P *= 0.05). Differences in nuclear positivity in AAC patients (50%) compared with control individuals or in AAC patients compared with ACC patients were not statistically significant (*P *= 0.33 and *P *= 0.34, respectively). There were no significant differences in distribution of cytoplasmic staining reactions between the different groups. Slides were scored in a two-scale system according to the intensity and extent of cytoplasmic and nuclear staining. According to this grading system, intense HIF-1α staining was observed in 57% (16/28) of AAC, in 100% (20/20) of ACC and in 44% (4/9) of control specimens (*P *< 0.001).

There was a statistically significant positive correlation between apoptosis and cell proliferation in ACC (*r *= 0.56, *P *= 0.016). The correlations between apoptosis and cell proliferation in the AAC (*r *= -0.16, *P *= 0.41) and control groups (*r *= 0.017, *P *= 0.97) were not statistically significant.

**Figure 2:**
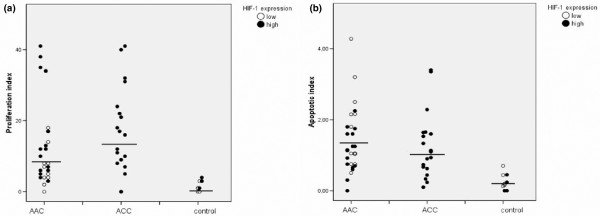
Epithelial cell proliferation and apoptosis. Provided are scatter plots showing epithelial cell **(a) **proliferation and **(b) **apoptosis in acute acalculous cholecystitis (AAX), acute calculous cholecystitis (AXX) and normal gallbladder. In each group, cases with a high hypoxia-inducible factor (HIF)-1α expression are shown with solid circles and those with low HIF-1α expression with open circles. Line indicates the median for each group.

In cases in which HIF-1α expression was low (score = 1), the cell proliferation rate was markedly lower (4.0% [1.0% to 8.0%]) than in cases with more intense HIF-1α staining (score = 2; 12% [5.50% to 26.50%], *P *= 0.002; Figure 2). There were no significant differences in apoptotic indices between the two HIF-1α groups; for cases with HIF-1α score of 1, the apoptotic index was 1.03% (0.47% to 2.16%) and for cases with HIF-1α score of 2, the apoptotic index was 0.93% (0.45% to 1.60%; *P *= 0.67).

The proliferation to apoptosis ratio tended to be higher in ACC (13.3 [6.3 to 27.0]) than in AAC patients (5.6 [3.2 to 19.9], *P *= 0.149) and control individuals (6.7 [0.7 to 15.8], *P *= 0.362). Furthermore, the proliferation to apoptosis ratio was significantly greater in the group with intense HIF-1α expression (13.7 [6.1 to 27.8]) than in the group with weak HIF-1α expression (3.7 [0.8 to 7.8], *P *= 0.001).

## Discussion

The present study shows that cellular proliferation and apoptosis were increased in AAC and ACC compared with normal gallbladders, indicating a higher cellular turnover rate, whereas the expression of HIF-1α was stronger in ACC than in AAC. On the other hand, the apoptosis rate in normal gallbladder epithelium was low.

To date, this study is the first to compare the gallbladder epithelial cell response to systemic inflammatory (AAC) or local inflammatory disease processes (ACC). Our previous histological and immunohistochemical studies have shown distinctive pathophysiological backgrounds in these two disease states [10,21]. Those studies and the present one provide evidence that both necrosis and apoptosis are involved in the destruction of gallbladder epithelium in both ACC and AAC. However, the extent of apoptosis is greater than that of necrosis in both types of cholecystitis. Although cell proliferation and apoptosis were increased compared with normal biliary mucosa, there were no differences between AAC and ACC.

In our series, apoptosis was significantly increased in AAC compared with control specimens. Apoptosis is a controlled process that is initiated through the expression of an endogenous cell death programme that requires sequential and co-ordinated action of various intracellular enzymes, which is modulated by HIF-1 and apoptosis-related genes [22]. Ischaemia-reperfusion injury has been suggested to be central to the pathogenesis of AAC, which has been shown to be associated with systemic sepsis, visceral arterial hypoperfusion and multiple organ dysfunction syndrome [3,4]. In rat models apoptosis has been found to be a major mode of cell death in ischaemia and ischaemia-reperfusion injury in the intestinal epithelium [13]. On the other hand, purulent inflammation and, frequently, bacterial infection are typical of ACC [3,9,10]. Apoptosis was also increased in ACC, which increase may have been triggered by a local neutrophil reaction, as has been demonstrated in intestinal epithelial cells [23].

The proliferation rate and the proliferation to apoptosis ratio appeared to be higher in the ACC than in the AAC group. HIF-1α expression was significantly higher in ACC samples. HIF-1α promotes cellular proliferation by inducing several growth factors [18,24]. Accordingly, the proliferation to apoptosis ratio was significantly higher in the samples with intense HIF-1α expression. It may well be that the stronger HIF expression accounts for the better preserved epithelial regeneration of the ACC mucosa. In murine experimental colitis, decreased HIF-1 expression correlated with more severe clinical symptoms, whereas increased levels were protective of mucosal epithelial barrier integrity [25]. Our previous studies also suggest significant differences in the integrity of gallbladder epithelia between AAC and ACC. Bile infiltration in the gallbladder mucosa is more abundant and extends deeper in AAC than in ACC [10], and there are differences in tight junction protein expression between ACC and AAC, which are important for epithelial barrier function [21].

The explanations for decreased HIF-1α expression in AAC are not clear. In a murine model, HIF expression is only sustained for a few hours in a hypoxic environment, implying that factors other than hypoxia are necessary for sustained elevation of HIF levels [26]. It has been suggested that over-expression is due to induction by cytokines released from inflammatory cells rather than hypoxia [27]. In addition, in human ischaemic colitis, HIF-1α is over-expressed in acutely ischaemic lesions compared with normal epithelia, and the expression is normalized after tissue recovery [28]. It could thus be speculated that, being an ischaemia-reperfusion phenomenon, HIF release is also time dependent and short-lived in AAC. Furthermore, during ischaemia and ischaemia-reperfusion, cell destruction and repair are affected in the various phases of the injury, and repeated ischaemia may also reduce epithelial apoptosis [29]. It should be noted that our patients were in different phases of their disease, which certainly could have affected the correlation of the different modulators.

In a rat model of gut ischaemia-reperfusion, HIF-1α activation rapidly disappeared on subsequent re-oxygenation, and this response was potentiated and preserved by the presence of bacteria or lipopolysaccharide [30]. This also agrees well with our results, which demonstrated stronger HIF-1α expression in ACC, which is associated with the intense mucosal neutrophil reaction. More than half of the gallbladders in the ACC group had bacterial growth, in contrast to only 7% in the AAC group. The histopathology of ACC is clearly a purulent local inflammation of the gallbladder. This further supports the notion that, in addition to inflammatory cells, the presence of bacteria potentiates the HIF response in ACC, keeping up the corrective cellular proliferation of the biliary mucosa. In addition, proliferation activity has been shown to be increased by gallbladder distension, which is also typical for ACC [31].

## Conclusion

In conclusion, cellular proliferation and apoptosis were increased in AAC and ACC, indicating higher cellular turnover compared with normal gallbladders. However, the expression of HIF was stronger in ACC than in AAC. These differences are probably accounted for by a local purulent inflammation of the gallbladder in ACC and systemic inflammation with visceral hypoperfusion in AAC.

## Key messages

• Cellular proliferation and apoptosis are increased in AAC and ACC compared with normal gallbladder.

• Expression of HIF-1α is lower in AAC than in ACC.

• Intense expression of HIF-1α is associated with increased cell proliferation.

## Abbreviations

AAC = acute acalculous cholecystitis; ACC = acute calculous cholecystitis; HIF = hypoxia-inducible factor; ICU = intensive care unit; SD = standard deviation.

## Competing interests

The authors declare that they have no competing interests.

## Authors' contributions

VM conducted the immunohistochemical assessments and participated in drafting of the manuscript. LJ participated in designing of the study, acquisition of data, analysis and interpretation of the results, and drafting of the manuscript. SJ contributed to designing of the study, acquisition of data, and analysis and interpretation of the results. KV contributed to designing and acquisition of data, and analysis and interpretation of the results. SH participated in designing and analysis, and interpretation of the data, as well as in critical revision of the manuscript. KT participated in the immunohistochemical analysis and in drafting of the manuscript. SY participated in the immunohistochemical analysis and in the critical revision of the manuscript. AKT participated in designing and coordination and in drafting of the manuscript, as well as in critical revision of the manuscript. All authors read and approved the final manuscript.
